# Random Thoughts on Our Specialty

**Published:** 1895-01

**Authors:** A. E. Baldwin

**Affiliations:** Chicago, Ills.


### Random Thoughts on our Specialty.

Read in the Section on Dental and Oral Surgery, at the Forty-fifth Annual Meeting of the American Medical Association, held at San Francisco, June 5-8, 1894.

BY A. E. BALDWIN, M.D., D.D.S., CHICAGO, ILLS.

Under this heading the writer feels free to wield a free lance, and he also feels that there are many things that may be properly grouped under such a title, thus giving a writer an opportunity to suggest varied lines of thought with possibly not a very logical grouping of them. In taking a retrospective view of our professional work and advancement during the last score of years, one can not help a strong feeling of disappointment, in spite of the very extravagant claims of the average dentist. In such a retrospective glance we must keep in mind that the effort of all truly scientific minds is toward that which is strictly scientific—and all will concede that the nearer we approach that exact position, the more incumbent it becomes us to make our so-called scientific work truly so, by making it clearly demonstrable. To place our specialty in such a position requires us to develop a class of people in our branch of professional work who will ever and always *think for themselves* on any and all subjects. We can not mix to any extent with our fellows in our society meetings without being impressed with what seems to be a fact, that at least 95 per cent. of our fellow workers allow themselves to simply swallow any honeyed or less daintily arranged bolus prepared for them by the other 5 per cent., and presented to them in the name of science in a grandiloquent way by one to whom, perhaps, had not yet been born a single original idea—or who knew not the difference between a "premise" and a "conclusion."

There just now occurs to the mind of the writer an illustration which he asks pardon for narrating. This occurred at a meeting of the American Dental Association a few years ago. One of our brethren, who *assumes* great dignity and profound knowledge, read a paper on "Carbolic Acid." The paper was lengthy and verbose, and was by many of the hearers hailed as a wonderful production; they almost made themselves appear as sycophants in their loudly expressed remarks in adoration of the wonderful ability displayed by the writer in his profound experimental research, as demonstrated by the paper; and doubtless many a young man hearing this paper and the comments of others upon it, went away from the meeting prepared to gulp down anything they might hereafter see emanating from his or his secretary's pen—when to strip this paper from its "ego" and husks you will find it simply a *compilation* from old authors, good and bad, and containing absolutely nothing of any practical or scientific importance but what was from some work direct; not a thought original with the author. The writer went away humiliated that among the many able men there gathered, there were so few who realized the imposition. Again, at another time, at a meeting of the Chicago Dental Society, the writer heard a so-called leader in our ranks—and this only a few years ago—say that he could always diagnose uterine diseases (presumably of women, though he did not say so) by the condition of the mouth of the patient, and the statement went almost unchallenged. What weight could be put upon anything said by such a "pseudo" scientific physician.

These illustrations are made, not for the purpose of casting slurs, but to call attention to the lack of exercise of discriminating power. What is uppermost in the writer's heart, is that the very small number of our brethren who will think for themselves, and not say "amen" to anything simply because some professor says so—but will weigh with physiologic, anatomic, histologic, pathologic or etiologic scales these so-called facts and then indorse or condemn as the scales may determine, that the number of these may increase and multiply until it will be very dangerous for one to stand up before them, in public or private, and feed them chaff, calling it wheat, with the hope that the statement will pass unquestioned.

Why should we be blind leaders of the blind, or allow the blind to lead us, though they may *claim* they see. We should be awake and learn to think for ourselves. Probably we, in our busy lives, may not have the time or opportunity for as much original investigation as we would wish, but this does not necessarily imply that we may arrive at the same conclusions that an original investigator does, for we (if he lucidly explains all the processes of the experiments) are in possession of all the "premises" from which he draws his "conclusions," and may view them far differently than he does.

In glancing over our journals the same thing is noticed—the immature thought is stated often as a certain conclusion and accepted as such. Many a specialty journal if put into the retort and subjected to the fire of scientific reasoning would come out wholly ashes, and many more would leave only the microscopic atom of truth.

If we might have some power given us to secure a journal that would contain, in each issue, nothing but demonstrable truths or plain, honest premises, from which to form our own conclusions, how small and choice such a work would be. Our shelves would never get overloaded, neither our minds overburdened in separating the gold from the dross. Then again we think all observing persons are aware that there are many in our ranks who are oftentimes satisfied to do, under certain circumstances, less than the best they can do. This is a matter that vitally concerns us all, for to slight an operation is to bring reproach upon ourselves as a whole. There is, of necessity, a great deal of difference in the various methods as practiced by different operators, but the rule that should govern in these matters is that we should not vary from well-established rules of operation unless we have well grounded physiologic reasons for believing the experimental method is an advance upon the old.

How few among us know the reason each time for what they do, or even try to solve the problem. How hard we have to drum the idea into the head of the average person, that growth or development of the part is induced by systematic exercise of the part, and if we as specialists expect a healthy and generous growth in mental and physical ability, it must come very largely, at least, not only by systematic thought and close study of books, but by training our powers of discrimination, so as to be able to determine the true from the false. Only a few days ago the writer had occasion to extract a tooth for a patient, in which he found that the pulp-chamber had been filled with, apparently, no attempt to clean the *debris* from the root canals or to fill them. Upon learning who the operator was and thinking it was a mistake on his part, he took occasion in the kindest way possible to call upon the dentist and show him the work and call attention to the history of the case; when, surprising as it may seem, the dentist said he knew he did not fill the root canals, and as an excuse said, for what he charged he could not afford to do any more work than he did.

Is it any wonder that when such things are done the people are distrustful of the ability or the honor of our workers? No one can afford to do less than the very best that lies in his power, regardless as to whether he gets a large price or does the work for nothing. The rule is called "Golden," that we should follow in this and in all matters; then the world, as well as our profession, would always be the better for our having lived.

